# Dispensing of Ivermectin From Veterans Administration Pharmacies During the COVID-19 Pandemic

**DOI:** 10.1001/jamanetworkopen.2022.54859

**Published:** 2023-02-01

**Authors:** Nora V. Becker, Sarah Seelye, Kao-Ping Chua, Kelly Echevarria, Rena M. Conti, Hallie C. Prescott

**Affiliations:** 1 Division of General Medicine, Department of Internal Medicine, University of Michigan, Ann Arbor; 2 VA Center for Clinical Management Research, Ann Arbor, Michigan; 3 Department of Pediatrics, University of Michigan, Ann Arbor; 4 Veterans Health Administration Pharmacy Benefits Management, San Antonio, Texas; 5 Questrom School of Business, Boston University, Boston, Massachusetts

## Abstract

This cohort study compares changes in ivermectin dispensing during the COVID-19 pandemic between the Veterans Administration (VA) and retail pharmacy settings and examines the association of the VA national formulary restriction with ivermectin dispensing.

## Introduction

Despite the lack of evidence that ivermectin is effective for SARS-CoV-2 infection,^1^ dispensing of ivermectin in US retail pharmacies increased sharply during the COVID-19 pandemic.^2^ This potentially harmed patients while resulting in wasteful insurer spending.^3^ Whether ivermectin dispensing also increased in the Veterans Administration (VA) is unknown. The VA has a national formulary that may discourage prescribing of ineffective drugs.^4,5^ In September 2021, the VA created national criteria that limited ivermectin use to parasitic infections but allowed adjudication on a case-by-case basis, according to local facility policy.^6^ The objectives of this cohort study were to compare changes in ivermectin dispensing during the COVID-19 pandemic between the VA and retail pharmacy settings and to examine the association of the VA national formulary restriction with ivermectin dispensing.

## Methods

This study was reviewed by the VA Ann Arbor institutional review board and was deemed exempt from the need for consent under 45 CFR §46, category 4 (secondary use of identifiable data). This study was reported according to the Strengthening the Reporting of Observational Studies in Epidemiology (STROBE) reporting guideline for cohort studies. The eAppendix in Supplement 1 includes additional methodologic details.

Data on ivermectin prescriptions dispensed at VA pharmacies from June 2019 to February 2022 were extracted from the VA’s Corporate Data Warehouse. We calculated the monthly ivermectin dispensing count per 100 000 active annual veterans, defined as any veteran with a hospitalization or filled medication.

Data on ivermectin prescriptions dispensed in US retail pharmacies were obtained from the IQVIA National Prescription Audit, which captures 92% of prescriptions dispensed in these pharmacies. Counts are projected to national totals. We calculated the monthly ivermectin dispensing count per 100 000 US residents.

Using a comparative interrupted time series design and ivermectin dispensing rates from June 2019 to February 2022, we assessed for differences in level and slope changes in monthly ivermectin dispensing rates after March 2020 between VA and retail pharmacies. To examine the association between the VA formulary restriction and ivermectin dispensing, we conducted a single-group interrupted time series analysis using only VA ivermectin dispensing rates without a control group, because we lacked data from a comparable population. The single-group analysis began in March 2020 and continued to February 2022, because ivermectin dispensing before March 2020 was infrequent. Data analyses were performed using Stata statistical software version 16.1 (StataCorp) and used 2-sided hypothesis tests with α = .05.

## Results

**Figure 1.**
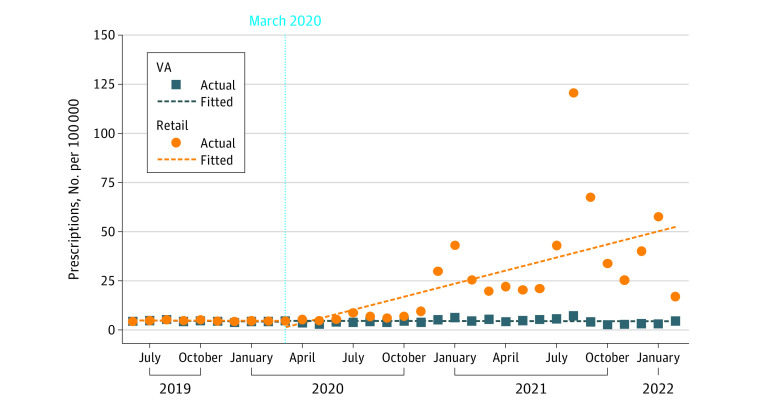
Ivermection Dispensing in Veterans Affairs (VA) and Retail Pharmacies Before and After March 2020 Monthly ivermectin dispensing counts per 100 000 people among VA and retail pharmacies from June 2019 through February 2022 are shown. The dotted blue line denotes the start of the COVID-19 pandemic in March 2020.

From June 2019 to February 2022, 7434 and 2 362 572 ivermectin prescriptions were dispensed by VA pharmacies and retail pharmacies, respectively. Before March 2020, monthly ivermectin dispensing rates were low and changed little in VA and retail pharmacies. After March 2020, the slope change in retail pharmacies was greater than the change in VA pharmacies (differential change, 2.17 additional prescriptions per 100 000 per month; 95% CI, 1.29-3.05 prescriptions per 100 000 per month) (Figure 1).

**Figure 2.**
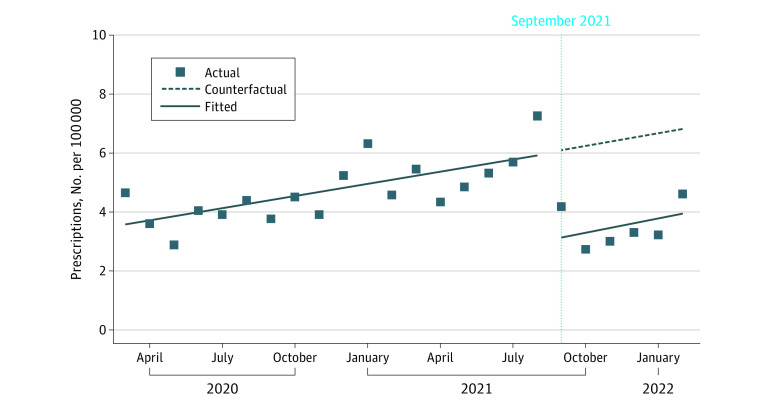
Ivermectin Dispensing in Veterans Affairs (VA) Pharmacies Before and After Formulary Restriction Monthly ivermectin dispensing counts per 100 000 active veterans in VA pharmacies from March 2020 through February 2022 are shown. The dotted blue line denotes the VA’s formulary restriction for ivermectin in September 2021.

From March 2020 through September 2021, the monthly ivermectin dispensing rate in VA pharmacies increased by 0.14 prescriptions per 100 000 per month (95% CI, 0.08 to 0.21 prescriptions per 100 000 per month). The formulary restriction was associated with an immediate level decrease (−2.83 prescriptions per 100 000; 95% CI, −3.80 to −1.86 prescriptions per 100 000) but no slope change (Figure 2).

## Discussion

This cohort study found that, in contrast to retail pharmacies, ivermectin dispensing in VA pharmacies increased minimally during the COVID-19 pandemic. Study limitations include lack of complete capture of all retail pharmacies in IQVIA data and the lack of a control group for the analysis of the VA formulary restriction.

Additional research is needed to determine why the surge in ivermectin dispensing seen in retail pharmacies did not occur in VA pharmacies. Local VA facility restrictions in ivermectin use may have existed before September 2021. Another possibility is that inappropriate demand for ivermectin increased less among veterans compared with nonveterans. Distinguishing between these possibilities could inform the design of interventions to decrease future provision of low-value care for COVID-19 and other conditions.

The VA formulary restriction implemented in September 2021 was also associated with an immediate decline in ivermectin dispensing. These findings highlight the ability of coverage restrictions to reduce the use of ineffective care.^3^ Such restrictions should be implemented carefully to avoid unintended consequences for patient access.

## Notes

Supplement 1. **eAppendix.** Additional Methodologic Details

Supplement 2.
Data Sharing Statement
