# Supplementary material for: Dispensing of Ivermectin From Veterans Administration Pharmacies During the COVID-19 Pandemic
Source: JAMA Netw Open. 2023 Feb 1;6(2):e2254859. doi: 10.1001/jamanetworkopen.2022.54859 (PMC9892958; doi:10.1001/jamanetworkopen.2022.54859)
Supplement: Supplement 1. — eAppendix. Additional Methodologic Details [file jamanetwopen-e2254859-s001.pdf]

Supplemental Online Content

Becker NV, Seelye S, Chua KP, Echevarria K, Conti RM, Prescott HC. Dispensing of ivermectin from Veterans Administration pharmacies during the COVID-19 pandemic. *JAMA Netw Open*. 2023;6(2):e2254859. doi:10.1001/jamanetworkopen.2022.54859

eAppendix. Additional Methodologic Details

This supplemental material has been provided by the authors to give readers additional information about their work.

© 2023 Becker NV et al. *JAMA Network Open.*

eAppendix: Additional Methodologic Details

<u>IQVIA National Prescription Audit:</u>
The IQVIA National Prescription Audit includes monthly dispensing counts from approximately 92% of U.S. retail pharmacies, 70% of mail-order pharmacies, and 70% of long-term care pharmacies. The latter two types of pharmacies accounted for minimal ivermectin dispensing during the study period, so results from this database most strongly reflect dispensing in the retail pharmacy setting. The database includes all prescriptions dispensed from participating pharmacies regardless of method of payment, including cash. Counts are projected to national totals using IQVIA's proprietary methodology.

<u>Estimates of the number of active Veterans:</u>
To estimate the total number of Veterans actively receiving care at the VA, the authors summed the annual total number of Veterans who filled a prescription at a VA pharmacy or had a hospitalization at a VA facility in a given calendar year. These data were derived from the VA Corporate Data Warehouse. This definition was chosen to avoid including Veterans who receive no care at the VA, as well as Veterans who only use the VA for extremely limited medical care that does not require contact with a VA provider (e.g., to obtain durable medical equipment or prescription glasses).

<u>National population estimates:</u>
The study period includes two different decades. Consequently, our analysis used estimates of the size of the U.S. resident population based on the 2010 U.S. Census (for data year 2019) and the 2020 U.S. Census (for data years 2020-2022).

The 2019 U.S. population estimates based on the 2010 Census may have underestimated the actual population count in those years. For example, on the basis of the 2010 U.S. Census, the Census Bureau estimated that there were 329,877,505 U.S. residents in July 2020. However, on the basis of the 2020 U.S. Census, the Census Bureau estimated that there were 331,501,080 U.S. residents in July 2020, or 1.004921751 times higher compared with the estimate based on the 2010 Census. We therefore multiplied the 2019 population estimate based on the 2010 U.S. Census by 1.004921751 to obtain a population estimate of 329,855,036.

The table below displays the annual denominators used for the calculation of ivermectin prescription rates for the VA and non-VA populations. The 2021 value was used as the denominator when calculating ivermection prescription rates during January and February 2022.

Of note, Veterans can also receive medications from retail pharmacies in addition to VA pharmacies, and so we chose not to subtract active Veterans from the U.S. population when calculating ivermectin dispensing rates for retail pharmacies.

**Year**	**Total U.S. population**	**Total active veterans**
2019	329,855,036	5,090,997
2020	331,501,080	4,976,678
2021 and 2022	331,893,745	4,999,883

© 2023 Becker NV et al. *JAMA Network Open.*

Statistical analyses:
We fitted two interrupted time series (ITS) models: 1) a comparative interrupted time series (CITS) model to compare differences in level and slope changes in ivermectin dispensing rates in March 2020; and 2) a single-group ITS model to assess for changes in dispensing rates per 100,000 within the VA after the formulary restriction on ivermectin was implemented in September 2021. The CITS model used ivermectin dispensing rates from June 2019 through February 2022 in both VA and retail pharmacies, while the ITS model used ivermectin dispensing rates in VA pharmacies from March 2020 through February 2022. The single-group ITS analysis did not include a control group, as pre-September 2021 trends in ivermectin dispensing at retail pharmacies diverged greatly from the VA and therefore could not be used as a comparable control group. After assessing for auto-correlation in both models, we used robust Newey-West standard errors with 10 lags for the CITS model and 4 lags for the ITS model. All analyses were performed using Stata statistical software, version 16.1 (StataCorp LLC) and employed two-sided hypothesis tests with $\alpha = 0.05$.

Additional details regarding the VA National Formulary (VANF):
The VANF Committee designates all drugs and drug-related supplies submitted for formulary consideration as either formulary or non-formulary. A subset of formulary agents, as determined by the Committee, is designated as prior authorization drugs requiring review and approval through the following established processes prior to dispensing.

Prior authorization determinations are completed at the national, Veterans Integrated Service Networks (VISN) or VA medical facility level, depending on the established prior authorization designation (i.e., PA-N, PA-V or PA-F). All formulary agents designated as prior authorization require Prior Authorization Drug Request (PADR) approval prior to dispensing. VA medical facilities may use multiple options for implementing PA-F requirements such as PADR consult, local order sets or drug file messaging.

In the absence of national guidelines, reasonable restrictions may be imposed at the VISN level. In some instances, it may also be appropriate for VISNs to further institute VA medical facility-specific restrictions; however, those restrictions must be clinically driven. National restrictions such as criteria-for-use and prior authorization criteria may not be altered by the VISN or VA medical facility. VISN restrictions must be evidence-based and allow for prescribing by authorized VA providers (with recognized expertise) when clinical conditions warrant their use. Restrictions may be based on economic issues if safety and efficacy are equivalent. Restrictions must not be so limited that patients with legitimate medical needs are prevented from receiving needed medications.

© 2023 Becker NV et al. *JAMA Network Open.*
