# Supplementary material for: Dispensing of Ivermectin From Veterans Administration Pharmacies During the COVID-19 Pandemic
Source: JAMA Netw Open. 2023 Feb 1;6(2):e2254859. doi: 10.1001/jamanetworkopen.2022.54859 (PMC9892958; doi:10.1001/jamanetworkopen.2022.54859)
Supplement: Supplement 2. — Data Sharing Statement [file jamanetwopen-e2254859-s002.pdf]

Data Sharing Statement

Becker. Dispensing of Ivermectin From Veterans Administration Pharmacies During the COVID-19 Pandemic. *JAMA Netw Open*. Published February 01, 2023. doi:10.1001/jamanetworkopen.2022.54859

Data
Data available: No
